# Bisphenol S in Food Causes Hormonal and Obesogenic Effects Comparable to or Worse than Bisphenol A: A Literature Review

**DOI:** 10.3390/nu12020532

**Published:** 2020-02-19

**Authors:** Michael Thoene, Ewa Dzika, Slawomir Gonkowski, Joanna Wojtkiewicz

**Affiliations:** 1 Department of Medical Biology, Faculty of Health Sciences, University of Warmia and Mazury in Olsztyn, Żołnierska 14C str., 10-561 Olsztyn, Poland; e.dzika@uwm.edu.pl; 2 Department of Clinical Physiology, Faculty of Veterinary Medicine, University of Warmia and Mazury in Olsztyn, Oczapowskiego Str. 13, 10-718 Olsztyn, Poland; slawomir.gonkowski@uwm.edu.pl; 3 Department of Pathophysiology, School of Medicine, University of Warmia and Mazury, 10-082 Olsztyn, Poland; Joanna.Wojtkiewicz@uwm.edu.pl

**Keywords:** bisphenol analogues, food packaging, obesogenic effects, metabolic disorders

## Abstract

In recent years, bisphenol analogues such as bisphenol S (BPS) have come to replace bisphenol A in food packaging and food containers, since bisphenol A (BPA) has been shown to leach into food and water, causing numerous negative health effects. Unfortunately, little or no research was done to determine the safety of these BPA-free products before they were marketed to the public as a healthier alternative. The latest studies have shown that some of these bisphenol analogues may be even more harmful than the original BPA in some situations. This article used a literature survey to investigate the bisphenol analogue BPS and compare it to BPA and other analogues with regards to increased obesity, metabolic disorders, cancer, and reproductive defects; among others. It was found that BPS works via different pathways than does BPA while causing equivalent obesogenic effects, such as activating preadipocytes, and that BPS was correlated with metabolic disorders, such as gestational diabetes, that BPA was not correlated with. BPS was also shown to be more toxic to the reproductive system than BPA and was shown to hormonally promote certain breast cancers at the same rate as BPA. Therefore, a strong argument may be made to regulate BPS in exactly the same manner as BPA.

## 1. Introduction

Bisphenol compounds are found throughout the contemporary world in the form of plastics that are used extensively by consumers for food storage. These polymers are also widely used in the packaging of baby formula, baby bottles, the lining of canned food and drink, dental implants, and sales receipts. Mainly though, these particular plastics enter the diet when people microwave food in plastic food containers or eat and drink from plastics that have been exposed to much wear or harsh chemicals that break down the monomers and release them into the food or drink. The use of bisphenols has been incredibly wide-spread and global for decades [1]. However, in 2007, the first indications were published that bisphenol polymers may leak monomers into food and drink and disrupt endocrine pathways by mimicking estrogen. Since then, BPA (bisphenol A) has become one of the most well-known EDCs (endocrine disrupting compounds) with pronounced effects on the reproductive system, child development, metabolic disorders, obesity, endocrine disorders, and the nervous system; as well as being implicated in causing DNA damage, oxidative stress, and breast cancer [2,3,4]. The concern about BPA affecting child development prompted a ban on BPA-containing products for babies in the EU, and guidelines to stop using BPA for baby bottles and formula packaging in the United States [5]. Therefore, the industry quickly formulated new BPA-free plastics to be used as food packaging and food storage containers instead. These BPA-free plastics are made using bisphenol analogs with very similar structural and chemical properties. For example, all bisphenols are manufactured by combining phenol with acetone (BPA), formaldehyde (BPF), hexafluoroacetone (BPAF) or sulfur trioxide (BPS). The latter monomer (BPS) is the most common bisphenol analog marketed as a BPA-free product [6], but there are quite a few other bisphenol analogs currently being sold as BPA-free products for food storage [7]. Bisphenols mainly enter the human body through contaminated food or water. However, contact with thermal paper in the form of sales receipts is also common, and those bisphenols may then enter the system via the handling of food [8].

Bisphenol S has been the most studied of the bisphenol analogues and is the most common substitute for BPA. The reasoning behind the substitution of BPS for BPA was that BPS was less likely to leach monomers into food and drink. This is not an unreasonable assumption, since BPS is generally more tolerant to heat and is more photo-resistant than BPA [9]. However, as BPS has become more common in society, there have been reports of as many as 81% of people in the United States and Asia testing positive for BPS exposure in their urine samples [10]. Therefore, over the last few years there has been a considerable scientific effort to evaluate the safety of BPS, since most people are being exposed to it in their diet.

Since bisphenol analogs are structurally and chemically similar to BPA, many researchers have recently been looking into the effects of these BPA alternatives. The study of BPA itself is relatively new, with the scientific community only reaching consensus that BPA is an EDC in 2013 [4]. The study of bisphenol analogs that have replaced BPA is an even newer field of study. For example, a review study from 2015 investigated all in vitro and in vivo studies of both BPS and BPF in the scientific literature up to that point and found only 32 articles. Only seven of those articles were in vivo studies [11]. A current database search of only BPS reveals several hundred studies that compare the bisphenol analog to BPA, and other BPA alternatives. The pathophysiological articles are mainly reporting correlations with cancer, metabolic disorders, obesity, DNA damage, liver damage, neural damage, oxidative stress, and most often endocrine effects and reproductive effects. The vast majority of these articles have been published between 2016 and 2019, with China and Europe taking the lead in this particular field of research. These publications also use many different types of tissue and cell cultures from mice to humans for in vitro studies. For in vivo studies, animal models from zebrafish to swine have been used, as well as clinical studies correlating bisphenol analogs with reproductive effects, mainly. 

## 2. Methods and Study Design

The aim of this article is to compare the pathophysiological effects of BPS with BPA and other bisphenol analogs through the use of a literature survey. Due to the structural similarities between BPA and BPS, there has been much recent research comparing the effects of BPS with the effects of BPA, and also between selected other bisphenol analogs. According to the literature, it seems that each bisphenol analog has differing toxicities depending on the organ system involved; however, the literature is quite consistent in reporting that all of the analogs of bisphenol are causing pathophysiologies as compared to controls. Since BPS is the most widely used analog currently in use, this article focuses on the toxicity of dietary exposure to BPS and compares it to controls and other forms of bisphenol. 

Using the electronic database from the University of Warmia and Mazury, it was possible to search across most academic database platforms and access articles from Pubmed/Medline, Elsevier, EbscoHost, Proquest, Scopus, Google Scholar, ResearchGate, and Web of Science. An advanced search function was used with the term “bisphenol S”. The results were limited to the category of “medicine”, with only peer-reviewed research articles being displayed. As a way of verifying the results, the above-mentioned database query was repeated with “BPS” replacing “bisphenol S”. The results of both searches were the same; however, the order of relevance was sometimes different. Only English language articles published in peer reviewed journals were included into the selection. Only articles that investigated a specific pathology with possible implications on human health were considered in the final analysis. Review articles were excluded so that results would not be duplicated, and only original laboratory or clinically based manuscripts were included. Since there was a previous review study of both bisphenol F and bisphenol S in 2015 (Rochester 2015), it was decided to only include articles that were published between 2015 and 2019 in order to review only the most recent studies. There were many articles describing how bisphenol compounds are manufactured, and those were excluded since they do not pertain to human health, per se. For this same reason, a database search using a CAS (registry number) to identify bisphenol S was not used, since those searches mainly are related to the manufacture of polymers and are not relevant to pathology, toxicology, or epidemiology. 

**Figure 1:**
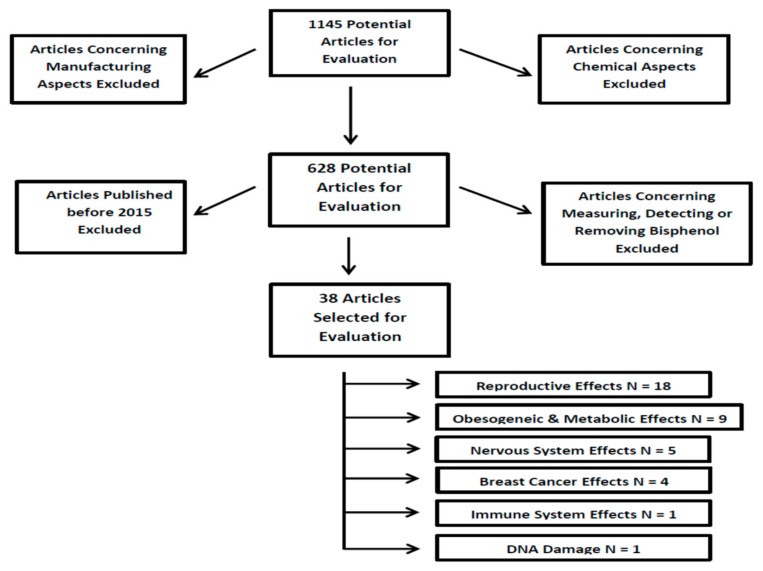
Flow chart of the article selection process. *N* = Population Size.

The database query described above returned numerous results. There were initially 1145 potentially relevant results. However, 517 of those results dealt with chemical aspects only, and they were excluded. Therefore, there were 628 results that were reviewed. The vast majority of those articles dealt with measuring or detecting BPS in humans or in foodstuffs, or they were concerned with methods of detecting or removing BPS from waterways. Those studies were excluded. Then, after only selecting articles published in 2015 or later, 38 articles remained detailing in vivo or in vitro pathological studies of the effects of bisphenol S with relevance to humans. For the purposes of quality control, two or more of the authors agreed on each of the final articles selected. There were six rough categories that all of the articles fell into. The 38 articles were subdivided into those categories for deeper analysis. There were 18 articles dealing with how dietary BPS exposure affects the reproductive system and/or the development of offspring in animal models. There were nine articles investigating dietary BPS and metabolic disorders, five looked into effects on the nervous system after dietary BPS exposure, four dealt with breast cancer, and there was one article each in the categories of dietary BPS and the immune system or damage to DNA. If overlap occurred between any subcategories, the main focus of the article was determined, and the article was placed into that subcategory. However, a discussion of the minor focus was also brought up in the appropriate subsection as well. Figure 1 provides a flow chart of the article selection process. 

## 3. Results

### 3.1. Dietary BPS Exposure and Reproduction

There were four articles from 2015, one from 2016, eight from 2017, three from 2018 and five from 2019 from a total of 21 articles concerning dietary BPS exposure and reproduction. Three of these articles were technically included under the sub-category of nervous systems, leaving 18 articles that studied the reproductive system exclusively. All of the studies analyzed the effects of BPS and other analogs on zebrafish, murines, bovine, swine, human cell lines, and in two clinical/observational trials. There were four zebrafish in vivo studies which reported mixed results. One study from 2017 tested the toxicity, estrogenicity, and teratogenic effects of BPA, BPS, BPF, and BPAF. Their results showed that the toxicity and estrogenicity were similar, with BPAF being more toxic and estrogenic than BPA; while BPS had the least effect. Meanwhile, they reported that teratogenic effects were observed in the heart, liver, fins, muscle, and certain endocrine organs. However, there was no mention of any neurological changes [12]. This is in contrast to another study done two years later, which also looked at the effects of BPA, BPS, BPF, and BPAF on zebrafish, and noted that all three damaged the developing nervous system. However, these two articles did agree exactly on the toxicity and teratogenic effects of the bisphenols. In both articles, the potencies were described as BPAF > BPA > BPF > BPS [13]. Therefore, both articles were in agreement that BPS was the least toxic to developing zebrafish. In 2016, one article was released that showed BPA and BPS both altered gene expression, which affected the reproductive neuroendocrine system of developing zebrafish. However, the article did not compare the potencies of the two bisphenols [14]. Meanwhile, there was a conflicting report from 2015 that reported BPS being more toxic than BPA during the neurogenesis of developing zebrafish. The neural development of the hypothalamus was observed, and the article reported a 180% increase in altered neurogenesis for BPA and a 240% increase for BPS [15]. While zebrafish may seem to be a poor model for what may be happening in humans after dietary bisphenol exposure, the authors referenced above cite that many of the genes and critical pathways for organ formation are highly conserved among these two species, with 70% of these human genes being found in zebrafish [12,13,14,15]. The last three studies mentioned above are also discussed in the section about the nervous system and were therefore not included into the count of eighteen reproductive system articles. Those three articles were counted under the topic of neurological effects, so as not to double count the overall number of articles. 

The most popular animal models for studying the effects of bisphenol S were mice, rats, or tissues derived from mice. There was a total of two in vitro and eight in vivo studies. Both of the in vitro studies reported that BPS and other bisphenols disrupted spermatogenesis. The paper from 2015 used murine MA-10 Leydig cells and exposed them to both BPS and BPF. Both bisphenol analogs increased progestagen levels and disrupted spermatogenesis. However, the article makes no comparison of the relative potencies. There is only the statement that both significantly decreased sperm production [16]. In agreement is an article from 2017, which used mouse spermatogonial cells and exposed them to BPS, BPA and BPAF. The authors reported that all three compounds exhibited spermatogonial toxicity, but BPAF was the most toxic. Furthermore, all of the bisphenols tested caused changes in the cell cycle, nuclear morphology, and disruptions of the cytoskeleton [17]. The in vivo studies were quite diverse and covered a wide range of reproductive topics from lactation to follicular development and from altered neural development to altered behaviors in mothers and offspring. In a 2017 article, mice were exposed to BPS during pregnancy and for the first 20 days of lactation. The mice showed normal mammary gland physiology at lactational day 2 (LD2), but at LD21, there was a significant decrease in the number of milk-producing lobules [18]. In the same year, another study was performed that included a study of the maternal behavior of mice after BPS exposure during pregnancy and lactation. That research showed that if mice are exposed to BPS in utero or during lactation, both the mother and the offspring have a higher probability of altered maternal behaviors [19]. In the same year, low dose exposure to BPS during early development was shown to alter the expression of estrogen-responsive genes, which caused the BPS-treated females to abnormally respond to an estrogen challenge later in life [20]. Other studies have shown altered neurogenesis and behavior when mice were exposed in utero to BPS, BPA, BPF, or BPAF [21,22]. Two articles specifically dealt with female infertility. The first documented a PCOS-like condition after exposure to BPS. Polycystic Ovary Syndrome (PCOS) has been known to cause infertility in humans. Several signaling pathways were involved, including the accumulation of a polarization marker in the cytoplasm of the BPS-treated groups [23]. The other article dealing with female infertility showed that BPS, BPA, and BPE all caused problems with the follicular development of mice. In all three groups treated with BPS, BPA, or BPE, there was a significant decrease in the rate of pregnancy, a decrease in the number of live births (with a corresponding increase in the number of dead pups), and more problems associated with giving birth to offspring [24]. Finally, there was another study published by the same group above that reported decreased sperm counts, sperm motility, and spermatogenesis caused by exposure to BPS, BPAS, and BPE disrupting sperm cell development. Interestingly, all three bisphenols caused a disruption in spermatogenesis, but only BPS decreased sperm motility [25].

Within the time-frame studied, there were two articles that dealt with bovine or swine models; one of each. In 2018, an article was published documenting the use of bovine oocytes exposed to both BPS and BPA. The authors found that both of the bisphenols caused significant abnormalities in the spindle formation along with chromosomal misalignment. However, BPS showed a higher incidence of causing both, leading to the conclusion that BPS may have more pronounced effects on oocytes [26]. The porcine study is more recent and looked at how BPS exposure affected cultured swine granulosa cells. Granulosa cells are the prime ovarian targets for bisphenols, and this may cause reproductive toxicity. This study found that BPS inhibited cell proliferation as well as nonenzymatic scavenging activity [27]. 

There were five studies done with regard to human cell lines/explants and clinical studies. Two of the in vitro studies used human testis explants to investigate the effect of several bisphenols on spermatogenesis. The first article was published in 2015, and examined BPS, BPA, and BPF exposure on human fetal testis. While all three decreased spermatogenesis, BPS exhibited its effects at a dosage ten times lower than BPA or BPF [28]. The second in vitro study was published in 2017, and also examined BPS, BPA, and BPF. This study used human testes explants taken from prostate cancer patients or multi-organ donors. Therefore, these were tissues from adult donors. The article reports that all three bisphenols inhibited spermatogenesis depending on concentration and the duration of exposure; however, the article did not note the relative potencies of the compounds against each other [29]. One study of human first trimester trophoblast cells determined that both BPA and BPS inhibit the early placentation process. The authors reported that this was due to the down-regulation of several genes, including the VEGF gene. This article also reported that BPA was more detrimental to both cell viability and growth than BPS [30]. The two clinical studies were conducted at hospitals/clinics and have been published within the last two years. The first article was a correlation study that recorded the preconception urinary levels of BPS and BPA. After birth, there were significant correlations between urinary BPA concentrations and lower birth weight and smaller head circumference. However, there were no consistent patterns between BPS urinary concentrations and these effects [31]. The final clinical article examined the correlation between several compounds, including BPS, and several serum hormones. Higher serum BPS levels were significantly correlated with lower corticotropin-releasing hormone (CRH) levels. CRH may have a role in triggering parturition. There were no other correlations that were relevant to bisphenol S in this particular article [32]. The results of Section 3.1 are summarized in Table 1 below. 

### 3.2. Dietary BPS Exposure and Obesogenic Effects/Metabolic Disorders

There was one article from 2015, three from 2016, four from 2017, none from 2018, and one from 2019 for a total of nine articles concerning dietary BPS exposure and obesogenic effects. The single article from 2015 indirectly studies BPS by exposing mice to BPS from the eighth day of pregnancy through postpartum day 21. The social interactions of the mice were then observed. This article will also briefly be discussed in the subsection concerning neurological changes after BPS exposure, but no neuronal pathologies were directly discussed in the article. Therefore, the article has been included into this subcategory. Curiously, the article reports a transient loss in body weight being associated with BPS. Most other articles have reported a correlation between all bisphenols and increased body weight [33]. However, this particular article was mainly interested in the effect of xenobiotics on the social behaviors of mice, the BPS correlation to decreased body weight seemed to be noted as a secondary effect, and the decreased weight was only temporary. In contrast to the above-mentioned decrease in body weight, there are three articles that were published the very next year that present exactly the opposite effect. All three articles document lipid accumulation in preadipocyte and adipocyte tissues. One article used primary human preadipocytes to examine the effect of BPS on adipogenesis. They found that lipid accumulation and protein levels for several adipogenic markers were increased after BPS exposure. That article also ran a series of experiments blocking several estrogen receptors, which then significantly blocked lipid accumulation. The study went on to propose that BPS is causing lipid accumulation and promoting the differentiation of preadipose cells via the peroxisome proliferator-activated receptor-γ (PPARG) pathway [34]. The second article of that year reported exactly the same results with regard to increased lipid accumulation and several increased adipogenic markers, but they used Murine 3T3-L1 preadipocytes. Furthermore, the study also found that BPS acted on the preadipocytes through the same (PPARG) pathway mentioned above. However, this article goes further by claiming that BPS caused the expression of more adipogenic markers than BPA, and that BPS follows a distinctly different pathway than BPA [35]. The final article from 2016 investigated the effect of BPA on pregnant mice. Pregnant mice were exposed to BPS in their drinking water through the entire pregnancy, and then the offspring were also given BPS via drinking water from birth to 23 weeks of age. BPS induced overweight mice, which was correlated to hyperleptinemia, hyperinsulinemia, and overall mass of fat. However, the results were limited to only mice that were fed a high fat diet as well as BPS. Their conclusion was that BPS helped to induce lipid storage during the course of a high fat diet [36]. An article from 2017 using pregnant sheep shows that gestational exposure to BPA and BPS may affect preadipocytes, but it affects them differently. BPA specifically caused adipogenic differentiation in females, but not in males. Meanwhile, BPS caused similar differentiation, but only in males [37]. Another team used human embryonic stem cells to build a model system to test obesogens. That team then used the system to test BPA and BPS. The exposed stem cell tissues increased their expression of adipogenic genes and also accumulated triglycerides [38]. The last article from 2017 was an observational study of children aged between 10 and 13 years. They found that urinary BPS levels were significantly correlated with insulin resistance, albuminuria, and irregular vascular function [39]. Finally, the single article from 2019 is an excellent large scale clinical/observational study from China. The study investigated gestational diabetes mellitus (GDM) among 1841 pregnant women and correlated the risk of GDM against urinary levels of BPS, BPA, BPF, and BPAF. By using multivariable logistic regression models, the researchers were able to show that high levels of urinary BPS and/or BPAF are correlated with GDM and may be a risk factor for developing gestational diabetes [40]. The results of Section 3.2 are summarized in Table 2 below.

### 3.3. Dietary BPS Exposure and Breast Cancer

In the time-frame reviewed here, there were four articles investigating dietary exposure to BPS and breast cancer. Three of those articles were published in 2017 and only one was published in 2019. Three of the four articles used MCF-7 human breast cancer cells, with one article using both MCF-7 and ZR-75-1 human breast cancer cells. The first article from 2017 tested BPAF, BPB, BPZ, BPA, BPF, BPAP and BPS on MCF-7 human breast cancer cells. They found that all of the bisphenol analogues acted as ERα agonists, but with varying estrogenic effects. The estrogenic potencies were evaluated as BPAF > BPB > BPZ = BPA > BPF = BPAP > BPS. The team then used microarray analysis to determine gene expression within the cell lines after bisphenol exposure, and stated that all of the bisphenols had altered the transcriptome of ERα containing cells. Their conclusion was that all of the bisphenol compounds helped to promote hormone-dependent breast cancer in varying degrees [41]. The next article from the same year also used MCF-7 human breast cancer cells, and focused on proliferation, epithelial mesenchymal transition (EMT) and migration after exposure to BPS, BPF and BPA. The study found that all three bisphenols induced cell proliferation, but they did not compare the potencies of the three compounds. Furthermore, with the use of Western blotting the study determined that all three compounds increased the expression of cyclin D1 as well as E1; both of which are cell cycle progression genes. The study also found that all three bisphenols caused cells to develop a fibroblast type of morphology, lose their cell adhesion and be more capable of migration after 24 h of exposure to the compounds. The researchers also found that during the increased capability for migration, all of the bisphenols caused N-cadherin protein expression to increase, while the expression of E-cadherin was decreased [42]. This is significant since E-cadherin is mainly found in epithelial cells, while N-cadherin is mainly found in mesenchymal cells. Other research has shown that this transition from E-cadherin to N-cadherin expression is a strong indicator of cancer progression [43]. The final article from 2017 is less technical than the previous two, but also comes to similar conclusions. The study used human primary adipocytes, rather than a cancer cell line, exposed them to BPS, BPF and BPA and assessed changes in the coding of the cellular RNA. They found that all three bisphenols caused an “enrichment” in pathways related to cancer progression. However, the potencies of the bisphenols relative to one another were not evaluated [44]. The final article dealing with cancer to be discussed is from 2019 and it used both MCF-7 and ZR-75-1 human breast cancer cell lines. The researchers examined whether several phenolic compounds would up-regulate the expression of aromatase mRNA within these cell lines, since this has been shown to cause the proliferation of certain breast carcinogens. Two of the phenolic compounds tested were BPS and BPA. The study showed that all of the phenolic compounds, including BPS and BPA, not only increased the expression of breast carcinogen, but that they also increased the proliferation of ERα positive breast cancer cells [45]. The results of Section 3.3 are summarized in Table 3 below. 

### 3.4. Dietary BPS Exposure and the Nervous System

Between 2015 and 2019, there were five articles investigating dietary exposure to BPS and pathologies of the nervous system. Three of those articles have previously been discussed in the sub-section on reproductive disorders; however, their main emphasis concerns the neural systems. Of these five articles, two were published in 2015, two in 2016, and only one was published in 2019. Four of the five articles used zebrafish as a model organism, with one article using C. elegans (nematodes). An honorable mention is also given to one article that looked at behavior patterns of mice after BPS administration. The article that employed mice as an animal model has been previously mentioned and is not counted into the statistics here. The findings were that mice exposed to BPS (among other endocrine disruptors) from the beginning of pregnancy through the 21st day after postpartum were less socially adept than control mice. The authors claim that the BPS treated mice displayed increased anxiety and were less interested in interacting socially [33]. The only other article in this sub-category that did not use zebrafish as a model used nematodes instead. C. elegans were exposed to BPS in early life, and their neural functionality was significantly impaired; persisting into adulthood. The researchers then compared these results to earlier work they had done with nematodes exposed to BPA and concluded that BPS and BPA followed a similar pattern of impaired neural functionality in nematodes [46]. The remaining articles all dealt with zebrafish. Most articles are in agreement and report similar results in some areas but contradict each other in other areas. Three out of four articles reported that bisphenols disrupted the hypothalamus during development. Two articles reported that BPS was the least toxic of all the bisphenols tested upon the nervous system, with one article claiming the exact opposite. An article from 2015 noted altered neurogenesis in the hypothalamus after developmental BPS and BPA exposure. The authors then stated that BPS caused more altered neurogenesis than BPA. The article then states that both bisphenols caused an increase in hyperactivity, but there was no comparison between BPS and BPA in terms of hyperactive potency effects [15]. There were two articles from 2016 that both reported BPS and other bisphenols disrupting the hypothalamus. One article describes several potential pathways for how both BPA and BPS may disrupt the neuroendocrine system within the hypothalamus. However, the article does not compare the potencies of BPA and BPS. However, they do state that both had significant effects at low levels of exposure [14]. The second article from 2016 compared three bisphenol analogues, BPS, BPA, and BPF. They found that all three affected the development of the hypothalamus, and also proposed a pathway based upon gene-expression analysis. Although all three compounds had a similar effect, BPS was noted to only disrupt the hypothalamus at very high concentrations; as compared to BPA or BPF [47]. The final article to be discussed was recently published in 2019 and was also previously mentioned. This article compared several bisphenol analogues and ranked them according to their ability to cause changes in locomotor activity. Toxicity to each type of bisphenol was assessed after exposure at ten days post-fertilization. The potencies were reported as being BPAF > BPB > BPF = BPA > BPS [13]. Once again, BPS was shown to be the least toxic to the development of neuronal tissues. The results of Section 3.4 are summarized in Table 4 below.

### 3.5. Dietary BPS Exposure, DNA Damage, and the Immune System

The remaining two articles fit into their own unique sub-categories. Both articles are from 2018. The first article used peripheral blood mononuclear cells (PBMCs) and exposed them to BPS, BPA, BPF, or BPAF at varying concentrations and for various lengths of time. Then the level of oxidative damage to DNA bases was recorded. The authors state that all of them caused oxidative damage to both purines and pyrimidines, but purines were most strongly affected. Furthermore, BPAF caused the most damage, while BPS caused the least damage to DNA bases [48]. In the same year, a study was performed exposing primary human macrophages (PMA-differentiated-U937 cells) to BPS, BPA, and BPAF at various concentrations for up to 96 h. The macrophages were then tested for cell viability and the levels of 30 different cytokines were measured. The authors found that the order of toxicity was BPAF > BPA > BPS, in terms of cell viability. When the cytokines were measured, they found that BPS had a very minor effect on cytokine secretion as compared to BPAF or BPA [49]. The results of Section 3.5 are summarized in Table 5 below. 

## 4. Discussion

According to the literature, the intake of dietary BPS in the form of contaminated food and water is the main source of exposure [6]. Mainly, exposure to BPA and analogues such as BPS comes from microwaving food in plastic containers made from these materials, from using plastic bowls and cups that are worn out and may be leaching monomers, or even from tap water in areas where bisphenols were used to coat the inside of water pipes. This is not insignificant when well over 80 percent of the worldwide population has detectable traces of BPS in their urine, and 97 percent have detectable traces of BPA [10]. Therefore, dietary exposure to these very common plastics is a danger that we have only very recently begun to understand. Moreover, BPS and other bisphenol analogs being used as a replacement in food packaging have not been studied as much as BPA has been. According to the literature, each bisphenol analogue has different properties, modes of action and even toxicities. Some studies have even found some of these bisphenol A substitutes to perhaps have even worse negative effects than BPA [11]. In some categories, dietary BPS seems to be the least toxic of all the bisphenol compounds; yet in other subcategories it seems to be the most toxic. Interestingly, every report examined here does state that BPS caused pathological changes when compared to controls; without exception. Nearly every article made a statement in their conclusion or discussion section that BPS should have exactly the same restrictions placed upon it as BPA, and one article went so far as to call BPS a “regrettable substitution” [50]. 

In terms of dietary BPS exposure affecting the reproductive system, BPS decreased sperm motility while other bisphenol analogues had no effect. In addition, BPS was more likely than BPA to cause abnormalities in the formation of spindle assemblies and to cause chromosomal misalignments in bovine oocytes. BPS was also able to decrease spermatogenesis at a ten times lower concentration than other bisphenol analogues. Detectable BPS in blood serum was also correlated with lower CRH hormone levels, which could interfere with natural parturition. While it is true that BPAF showed a significantly higher level of spermatotoxicity, BPS was tied with BPA for being the second most spermatotoxic bisphenol analogue. Other articles that dealt specifically with the reproductive system simply stated that both BPA and BPS caused significant pathologies. Therefore, an argument could be made stating that BPS is more toxic to the reproductive system than BPA, and perhaps most other bisphenol analogues as well. However, far more research does need to be performed in order to verify such a hypothesis. 

With regards to the effect of dietary BPS on obesogenics and metabolic disorders, there was clear agreement that BPS caused lipid accumulation in adipocytes and preadipocytes, and that BPS acted via a different pathway than BPA. There was only a single article that reported BPS exposure causing mice to lose body weight; however, that report was not the primary focus of the study and the study did not look into the long-term weight gain of mice exposed to dietary BPS. Other reports that did look into the effect of long-term exposure to BPS found the opposite. All mice gained body weight, and that weight was found to be from fat tissue. However, the lipid accumulation was only correlated with mice fed with a high fat diet. The BPS, apparently, helped the mice to more easily activate the preadipocytes and store the excess lipids more easily. Moreover, BPS seemed to only activate preadipocytes in males, but not in females, which is exactly the opposite effect of BPA. BPA only activated preadipocytes in females, but not in males. Finally, a large clinical/observational study found that both detectable BPS and BPAF levels were significantly correlated with gestational diabetes (GDM), while other bisphenol analogues were not correlated. Therefore, an argument could be made stating that BPS is just as likely to cause obesogenic effects and metabolic disorders as BPA and most other bisphenol analogues. However, more research needs to be performed to clarify exactly the potency of BPS versus other bisphenols in terms of obesogenics and metabolic disorders. 

Dietary BPS has a demonstrable effect on human breast cancer. BPS was found to be an ERα agonist, as were all other bisphenols tested, which both increased the proliferation of the cancer cells as well as caused their migration. All of the articles noted that BPS did cause more proliferation and/or migration of cancer cells as compared to controls, but one article testing seven different bisphenols found BPS to be the least estrogenically potent. BPAF was the most potent promoter of breast cancer cell development, as compared to other bisphenols, with BPS the least active. However, compared to controls, BPS still significantly increased the development of ERα breast cancer cells. Therefore, an argument could be made stating that BPS is least likely to promote the proliferation and/or migration of breast cancer than most other bisphenol analogues. However, BPS is still an ERα agonist and promotes breast cancer, just not as much as other bisphenols. Far more research needs to be performed to further clarify this issue. 

Dietary BPS also has an effect on the nervous system, but its effects compared to the other bisphenols are conflicting in the literature. Of the studies, the ones that did not use zebrafish as a model simply showed that BPS causes pathologies. The zebrafish studies were more detailed, but conflicting. One study reported that BPS caused altered neurogenesis at a higher rate than BPA, but other studies using the same zebrafish models reported the exact opposite effect. One study showed BPS disrupted the hypothalamus, but only at relatively high concentrations, while another study looked at changes in locomotor activity after exposure to several bisphenols. They found that BPS was the least toxic, while BPAF was the most toxic. All articles did agree that BPS caused alterations in neural development when compared to controls, but they were in disagreement about the relative toxicity. In order to clarify the issue of relative toxicity, more research needs to be conducted. 

Dietary BPS exposure was found to be the least toxic in terms of causing oxidative damage to DNA in peripheral blood studies, with BPAF causing the most damage. BPS was also found to be the least likely bisphenol to damage the immune system, with BPAF causing the most damage. BPS was least likely to destroy macrophages and had a negligible role in causing cytokine secretion. This was in contrast to the pathologies caused by BPAF. 

## 5. Conclusions

In summary, dietary exposure to the BPA replacement known as bisphenol S, or BPS, is likely more toxic and seems to cause more pathologies in the reproductive system than the original BPA or any of the other BPA analogues. BPS had an effect that was equal to all of the other bisphenols in obesogenics and metabolic disorders, and the results were mixed as to BPS toxicity in the nervous system. Dietary exposure to BPS appeared to be the least likely to promote a certain type of breast cancer, cause DNA damage or weaken the immune system. However, in all cases BPS exposure did promote these pathological effects, as compared to controls. These results give strong evidence that each bisphenol analogue interacts differently in vivo, and that some bisphenol analogues may be more toxic to one system, while being less toxic to another system. Generally though, the authors of this study agree with the majority of the other authors reviewed here: BPS should be put under the same legal restrictions as BPA. 

## Figures and Tables

**Table 1:** Summary of dietary bisphenol S (BPS) exposure and reproduction.

Specific Effect	Model Used	Results	Reference Number
Reproductive System	Zebrafish	Mixed results with 3 studies claiming BPS was the least toxic to developing zebrafish and one study claiming BPS was the most toxic	Moreman et al. [12] Catron et al. [13] Qiu et al. [14] Kinch et al. [15]
	Murine	BPS and BPA decreased sperm counts, sperm quality, and spermatogenesis but only BPS decreased sperm motility. In females, BPS decreased lactation, disrupted follicular development, caused an abnormal estrogen response, increased female infertility and altered maternal behavior	Roelofs et al. [16] Liang et al. [17] Shi et al. [25] LaPlante et al. [18] Catanese & Vandenberg [19] Hill et al. [20] Castro et al. [21] Ohtani et al. [22] Demacopulo & Kreimann [23] Shi et al. [24]
	Bovine	BPS caused the most abnormal oocyte spindle formation & chromosomal misalignment	Campen et al. [26]
	Porcine	BPS inhibited cell proliferation & nonenzymatic scavenging activity causing reproductive toxicity	Berni et al. [27]
	human cell lines or explants	BPS more easily decreased spermatogenesis, BPA and BPS both inhibit early placentation	Eladek et al. [28] Desdoits-Lethimonier et al. [29] Basak & Duttaroy [30]
	Clinical/Observational Studies	BPS was not correlated with lower birth weight and smaller head circumference, but was correlated with lower Corticotropin-releasing hormone (CRH) levels	Mustieles et al. [31] Aker et al. [32]

**Table 2:** Summary of dietary BPS exposure and obesogenic effects/metabolic disorders.

Specific Effect	Model Used	Results	Reference Number
Obesogenic Effects/Metabolic Disorders	Murine	BPS caused a transient loss in body weight in one behavioral study, but caused increased body weight via the activation of preadipocytes in all other studies	Kim et al. [33] Boucher et al. [34] Achmed & Atlas [35] Ivry Del Moral et al. [36]
	Sheep	BPS caused adipogenic differentiation in males, while BPA had the same effect in females	Pu et al. [37]
	human embryonic stem cells	BPS increased cellular expression of adipogenic genes and caused accumulated triglycerides	Wang et al. [38]
	Clinical/Observational	BPS levels correlated with insulin resistance, albuminuria, irregular vascular function and gestational diabetes mellitus (GDM)	Kataria et al. [39] Zhang et al. [40]

**Table 3:** Summary of Dietary BPS Exposure and Breast Cancer.

Specific Effect	Model Used	Results	Reference Number
Human Breast Cancer	MCF-7 human breast cancer cells	BPS had the least effect on estrogen receptors (ERα), but did promote cancer progression	Mesnage et al. [41] Kim et al. [42] Gravdal et al. [43] Williams et al. [45]
	human primary adipocytes	BPS caused coding changes consistent with cancer progression	Verbanck et al. [44]

**Table 4:** Summary of dietary BPS exposure and the nervous system.

Specific Effect	Model Used	Results	Reference Number
Nervous System	Zebrafish	Mixed results with four studies reporting opposite findings concerning neural development after BPS exposure	Catron et al. [13] Qiu et al. [14] Kinch et al. [15] Cano-Nicolau et al. [47]
	C. elegans	BPS significantly impaired neural function	Mersha et al. [46]

**Table 5:** Summary of dietary BPS exposure, DNA damage, and the immune System.

Specific Effect	Model Used	Results	Reference Number
DNA Damage	peripheral blood cells (PBMCs)	BPS caused the least damage to DNA bases of all tested bisphenols	Mokra et al. [48]
Immune System Effects	primary human macrophages	BPS had a very minor effect on cytokine secretion	Chen et al. [49]

## Abbreviations

BPA	Bisphenol A
BPB	Bisphenol B
BPS	Bisphenol S
BPF	Bisphenol F
BPE	Bisphenol E
BPAF	Bisphenol AF
BPAP	Bisphenol AP
BPP	Bisphenol P
BPZ	Bisphenol Z
CRH	Corticotropin Releasing Hormone
GDM	Gestational Diabetes Mellitus
EMT	Epethelial Mesenchymal Transition
ERα	Estrogen Receptor alpha
LD	Lactation Day
PPARG	Peroxisome proliferator-activated receptor gamma
VEGF gene	vascular endothelial growth factor gene
